# Society Meetings

**Published:** 1903-07

**Authors:** 


### SOCIETY MEETINGS.

The Medical Society of the County of Chautauqua will hold its annual meeting at Chautauqua, N.Y., July 8, 1903, under the direction of and observing a program prepared by Dr. G. E. Blackham, of Dunkirk, N. Y., president of the society. The entire session will be devoted to the subject of tuberculosis under the following titles: Legislation concerning tuberculosis, Hon. Elton D. Warner, late Deputy Attorney General; Value and limitations of tuberculin test in cattle, Hon. Frank E. Shaw of the New York State Tuberculosis Commission; Report of a case of tubercular transmission from bovine to human, Dr. G. E. Smith, Cherry Creek; The etiology, Dr. G. E. Blackham; Bacteriological diagnosis, Dr. A. W. Dods, Fredonia; Treatment, drugs, climate, and the like, Dr. N. G. Richmond, Fredonia; *x*-Ray treatment, Dr. M. Moore, Fredonia. An excursion rate of $1.00 round trip from Buffalo, will make the place cheaply reached and afford a most delightful trip. The society extends to the profession of Buffalo and surrounding counties a most cordial invitation to be present and take an active part in the discussions.

---

THE American Electro-therapeutic Association will hold its thirteenth annual meeting at the Windsor Hotel, Atlantic City, Tuesday, Wednesday and Thursday, September 22, 23 and 24, 1903, under the presidency of Dr. Daniel Roberts Brower, Chicago.

---

THE Fourth Pan-American Medical Congress will be held in Buenos Aires, S. A., in 1905. The international executive committee at its meeting held April 1, 1903, decided to accept this amended date upon the proposal of the members from the Argentine Republic, to avoid a conflict of dates with other national and international associations and congresses.

---

THE Erie County Medical Association held its quarterly meeting at the University Club, Buffalo, Monday, June 8, 1903, at 8 p. m. under the presidency of Dr. Allen A. Jones.

*Program*:—The relation of common pelvic diseases to the general health, by Dr. C. C. Frederick. Discussion by Drs. Maud J. Frye, H. E. Hayd and Ray H. Johnson. Differential diagnosis of tumors of the breast, by Dr. E. J. Meyer. Discussion by Drs. Phelps, Kenerson, and Clinton. The relation of the physician to the puerperal woman and her child, by Dr. W. G. Taylor. Discussion by Drs. Huntley, Sherman and Trowbridge. Hemorrhage following tonsillotomy with report of a serious case, by Dr. Adolph H. Urban. Discussion by Drs. Renner, Cott and Meyer.

---

THE Fourth District Branch of the New York State Medical Association, held its nineteenth annual meeting, at the Buffalo Club, in this city, June 16, 1903.

The following program was observed: President's address, J. W. Morris, Jamestown; Intestinal obstruction, Wm. C. Phelps, Buffalo: discussion: Zera J. Lusk. Spurious gastric symptoms, Allen A. Jones, Buffalo; discussion: John G. Kelly. The radical operation for chronic suppurative otitis, Arthur B. Duel, New York; discussion: W. Scott Renner. Results obtained by prostatectomy, Parker Syms, New York; discussion: Wm. C. Phelps. The obstetric significance of retroversion and retroflexion of the uterus, Samuel M. Brickner, New York; discussion: C. C. Frederick. Hints on the hygiene and dietetics of children, H. F. Gillette, Cuba; discussion: Dewitt H. Sherman. Pseudomyelitis, Chas. R. Phillips, Hornellsville; discussion: Chas. A. Wall. A paper on smallpox, Wm. A. Macpherson, Leroy; discussion: Geo. A. Himmelsbach. Ocular incoordination and cerebral reflexes, F. Park Lewis, Buffalo; discussion: A. A. Hubbell.

Dr. John Herr Musser, of Philadelphia, president-elect of the American Medical Association, presented a paper on Cancer of the lungs.

---

THE Buffalo Academy of Medicine held its annual meeting Tuesday evening, June 9, 1903. Program: Election of officers; an address by the retiring president, Dr. Herman E. Hayd. Installation of newly elected officers.

---

THE American Association of Obstetricians and Gynecologists will hold its sixteenth annual meeting at Chicago, Tuesday, Wednesday and Thursday, September 22, 23 and 24, 1903, under the following administration: president, Lehman H. Dunning, Indianapolis; vice-president, Marcus Rosenwasser, Cleveland, and Herman E. Hayd, Buffalo; secretary, William Warren Potter, Buffalo; treasurer, Xavier O. Werder, Pittsburg; executive council, Edward J. Ill, William A. Humiston, Edwin Ricketts, Walter B. Chase, Albert Vander Veer and Lewis S. McMurtry. The preliminary program is in process of preparation to be sent out July 15, and it is desirable that the titles of papers to be read should be furnished the secretary promptly, in order to be included.

Dr. John B. Murphy, of Chicago, is the chairman of the committee of arrangements and will supply any information with reference to the local features of the meeting upon request.

